# Chitin- and Chitosan-Based Composite Materials

**DOI:** 10.3390/biomimetics7010001

**Published:** 2021-12-21

**Authors:** Inmaculada Aranaz, Niuris Acosta

**Affiliations:** 1 Instituto Pluridisciplinar de la UCM, Paseo Juan XXIII, n1, 28040 Madrid, Spain; 2 Department of Chemistry in Pharmaceutical Sciences, Faculty of Pharmacy, Complutense University of Madrid (UCM), 28040 Madrid, Spain

Chitin and its deacetylated derivative chitosan are amino polysaccharides of great interest due to their biological and technological properties. Chitosan composites using myriad components have been produced to improve chitosan-based materials (Figure 1). By selecting the appropriate additive, properties such as mechanical properties or water permeability can be improved, or new functionalities can be found. These materials find applications in packaging [1], drug delivery [2], tissue engineering [3], hyperthermia therapy [4], water pollutant removal [5], air purification [6] or fuel cell applications [7], among others.

In this Special Issue, several examples of chitosan-based composites are presented with different purposes.

Osorio-Madrazo and co-workers explore fibre-reinforced hydrogels to be used for the repair and regeneration of the intervertebral disc (IVD) annulus fibrosus (AF) tissue [8]. The choice of this composite is based on the similar microstructure, mechanical properties, and functionality of this composite with the biological tissue. In this work, the development of a new chitosan physical hydrogel filled with cellulose nanofibers (nano-fibrillated cellulose) to reinforce the chitosan matrix is presented. The implantation of the composites in AF disc defects during ex vivo experiments performed in pig vertebral unit models showed that the composites contribute to the restoration of the disc biomechanics by approaching the functionality of a healthy disc.

In the second contribution, chitosan composites are produced with polyaniline powders using a solution casting method [9]. The addition of polyaniline to the non-conductive chitosan adds a new property to the chitosan films. The composites showed good cell viability similar to that of chitosan films. This opens the use of this composite in biomedical applications in which the electrical properties of polyaniline are advantageous in comparison to non-conductive chitosan materials.

Access to safe drinking water is a fundamental need and human right. The treatment of wastewater is a serious concern, so materials designed to clear water are gaining large interest. It is well known that iron oxides are very potent sorbents for a variety of elements that include strontium. However, in solution at basic pH, they exhibit poor mechanical properties and stability. In the third paper, chitosan composites containing iron oxides are produced to eliminate Sr^2+^ ions from tap water and wastewater avoiding the drawbacks of iron oxides [10].

Metallic chitosan-based composites can be easily produced using chitosan both as a reducing and stabilizing agent. However, the control in the production of the nanoparticles depends on the physicochemical properties of the chitosan used. This effect is poorly studied in the literature and mainly ascribed to chitosan Mw and deacetylation degree. Aranaz and co-workers demonstrated that the chitosan pattern also affects the process of AgNPs production in terms of nanoparticle size and stability [11]. This will allow for the production of AgNPs composites in which it is possible to control nanoparticle size and thus controlling catalytical, optical, and antimicrobial properties.

In the last paper, Goycoolea and co-workers reviewed the interaction between mucins and chitosan in order to gain deeper knowledge of the mucoadhesive properties of chitosan [12]. This property is fundamental in the application of chitosan in the design of transmucosal drug delivery systems, as well as for the treatment of pathologies related to mucosal dysfunctions. Chitosan interaction with mucins not only depend on its acetylation degree, but also the pattern plays a fundamental role.

## Figures and Tables

**Figure 1:**
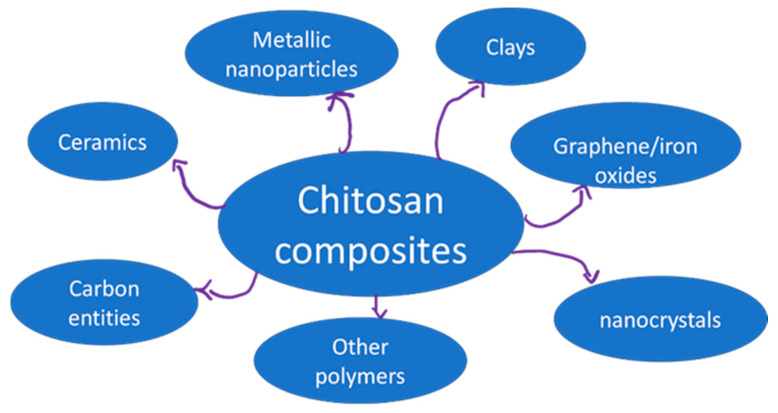
Types of components found on chitosan composites.

## Data Availability

Not applicable.
